# Proton beam therapy for cancer in the era of precision medicine

**DOI:** 10.1186/s13045-018-0683-4

**Published:** 2018-12-12

**Authors:** Man Hu, Liyang Jiang, Xiangli Cui, Jianguang Zhang, Jinming Yu

**Affiliations:** 1 Shandong Cancer Hospital Affiliated to Shandong University, Jinan, China; 2 Shandong Academy of Medical Sciences, Jinan, China; 3 Departments of Radiation Oncology and Shandong Province Key Laboratory of Radiation Oncology, Shandong Cancer Hospital and Institute, Jinan, China; 4 Province Key Laboratory of Medical Physics and Technology, Center of Medical Physics and Technology, Hefei Institutes of Physical Science, Chinese Academy of Sciences, Hefei, Anhui China; 5 Departments of Radiation Oncology, Zibo Wanjie Cancer Hospital, Zibo, Shandong China

## Abstract

Precision radiotherapy, which accurately delivers the dose on a tumor and confers little or no irradiation to the surrounding normal tissue and organs, results in maximum tumor control and decreases the toxicity to the utmost extent. Proton beam therapy (PBT) provides superior dose distributions and has a dosimetric advantage over photon beam therapy. Initially, the clinical practice and study of proton beam therapy focused on ocular tumor, skull base, paraspinal tumors (chondrosarcoma and chordoma), and unresectable sarcomas, which responded poorly when treated with photon radiotherapy. Then, it is widely regarded as an ideal mode for reirradiation and pediatrics due to reducing unwanted side effects by lessening the dose to normal tissue. During the past decade, the application of PBT has been rapidly increasing worldwide and gradually expanding for the treatment of various malignancies. However, to date, the role of PBT in clinical settings is still controversial, and there are considerable challenges in its application. We systematically review the latest advances of PBT and the challenges for patient treatment in the era of precision medicine.

## Background

**Fig. 1:**
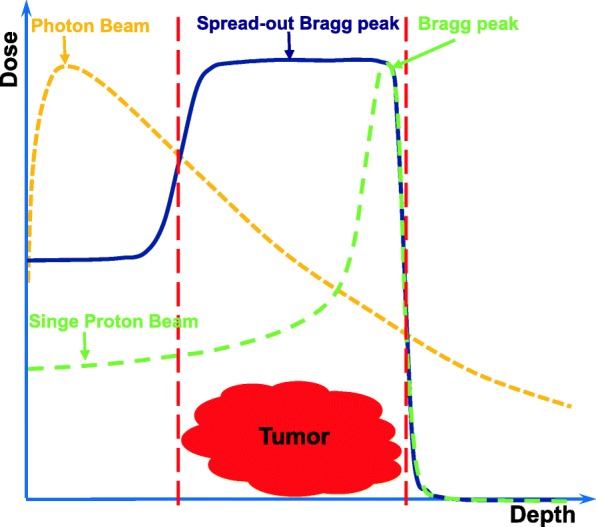
The diagram of dose distributions for photon (dashed yellow line), single proton beam (dashed green line) as a function of penetration depth in tumor (normalized to the maximum dose), and spread-out proton beam (solid blue line)

Radiotherapy (RT) is an established treatment modality of malignant tumors. Currently, photon beam therapy is the most widely used in clinical settings. Intensity-modulated photon radiotherapy (IMRT) was introduced in the mid-1990s, and it took the radiotherapy with photons to a huge leap forward. As the development of IMRT, it has been considered to be the advanced and the standard of treatment for many malignancies [1]. Although the IMRT technique can typically provide a more conformal dose distribution than the traditional RT mode, it is necessary to improve the tumor control and overall survival (OS), and reduce the RT toxicity. It is well known that the advantage of a proton beam is the physical characteristics of its depth-dose curve, with a dose peak (Bragg peak) at a well-defined depth in tissue (Fig. 1). For relatively shallow tumors, unlike the photon depth-dose curve showing an exponentially decreasing energy deposition with increasing depth in tissue, the Bragg peak allows for rapid fall-off of the radiation dose at the end of the range and a sharp lateral dose fall-off with the maximum energy deposition for each proton beam in the target region and almost no energy around it. Therefore, proton beam therapy (PBT) effectively allows the delivery of high-radiation doses to tumor cells and very low or zero doses to the normal cells, which is recognized as an ideal therapy modality for treatment of malignant diseases, especially for organs at risk (OARs) with less toxicity. As Dr. Herman Suit in the department of radiation oncology of Massachusetts General Hospital (MGH) said: “No advantage to any patient for any irradiation of any normal tissue exists; and radiation complication never occurs in nonirradiated tissues.”

In 1946, Robert R. Wilson proposed to use accelerator-produced beams of protons to treat patients with deep-seated tumors [2]. In 1954, the first patient with breast cancer was treated with proton radiation of the pituitary in the Berkeley Radiation Laboratory [3]. In 1961, protons commenced to be used for clinical treatment at Harvard Cyclotron Laboratory [4]. Initially, the clinical practice and research of PBT only focused on the tumors near a critical structure or those that responded poorly to photon radiotherapy such as ocular tumors, skull base tumors, paraspinal tumors, and unresectable sarcomas. Over the next 60 years, with the vast development of technology, the application of PBT has been gradually expanding to various neoplasms. Although increasingly more evidence has been indicated for the advantages of PBT in clinical experience, PBT is not good for all cases all of the time. It is very important to understand the benefits and limitations of protons as well as the biology and the behavior of the tumor. In this review, we summarized the latest advances and clinical applications of PBT. We also considered the challenges of treatment optimization in the era of precision medicine.

## Latest clinical studies of PBT

The dosimetry advantage of protons over photons has already been established (which is not reviewed in the article). However, do the potential advantages of the proton beam significantly transfer into clinical benefits for patients? Can the advanced techniques such as 360° rotational gantries and intensity-modulated proton therapy (IMPT) further minimize toxicity and/or improve the clinical outcome? To date, there is not enough evidence to answer these questions due to small cohorts of patients in most published studies and the limited prospective data of comparisons between proton and photon radiotherapy. In this part, we present the clinical experiences and studies in the past few years, which may be provide a valuable understanding of the true value and advantage of PBT.

### Reirradiation

Reirradiation may provide the best chance of long-term disease control and even a potential cure for the patients who truly undergo local and/or regional recurrence and who would not develop distant metastasis. The physical characteristics of PBT are particularly suited for reirradiation, which has been reported in head and neck cancer (HNC), thoracic cancers and liver cancer.

The largest report of recurrent HNC to date was an analysis of 92 patients treated with a proton beam using passive scatter technique reirradiation by Romesser et al. [5]. The median doses were 60.6 Gy, and the 1 year cumulative incidence of locoregional failure (LRF), actuarial freedom of distant metastasis (FDM), and overall survival (OS) were 25.1%, 84.0%, and 65.2%, respectively. Eighty-seven (94.6%) patients completed the reirradiation course. Acute grade ≥ 3 toxicities of mucositis, dysphagia, esophagitis, and dermatitis accounted for 9.9%, 9.1%, 9.1%, and 3.3%, respectively. Late grade ≥ 3 adverse events included skin (8.7%) and dysphagia (7.1%), and only two patients (2.2%) underwent grade 5 treatment-related bleeding toxicity. Phan et al. [6] evaluated 60 HNC patients receiving proton beam reirradiation. Twenty-five percent patients (15/60) received passive scatter proton therapy (PSPT), and 75% (45/60) received IMRT. The 1 year rates of locoregional failure-free survival (LRFFS), progression-free survival (PFS), OS, and distant metastasis-free survival (DMFS) were 68.4%, 60.1%, 83.8%, and 74.9%, respectively. Acute grade 3 toxicity occurred in 30% patients (18), and 22% (13) needed a feeding tube. The 1-year rates of late grade 3 toxicity and feeding tube independence were 16.7% and 2.0%, respectively. Three patients may have died due to reirradiation-related toxicity. For patients with recurrent HNC, it is safe and effective to reirradiate disease by utilizing proton beam, which has acceptable rates of complications and durable tumor control and survival.

Because more patients with non-small cell lung cancer (NSCLC) have better survival, recurrence can occur more often in the previously irradiated area or adjacent area. Earlier published studies had explored the role of proton beam reirradiation for recurrent NSCLC patients, and most were focused on the palliative intent with lower overall doses. Recently, with definitive intent, Chao et al. [7] have reported the safety/feasibility of PBT for locally recurrent NSCLC (*n* = 57) in a multi-center prospective study. More than 90% of patients completed the reirradiation course. With a median dose of 66.6 Gy, locoregional control (LRC) was 75%, with 1- and 2-year OS rates of 59% and 43%, PFS of 58% and 38%, respectively. Twenty-four patients (42%) developed grade ≥ 3 acute and/or late toxicities. Six patients experienced grade 5 toxicities. In the study, the proton plan was largely double-scatter (*n* = 34 [59.6%]) or uniform scanning (*n* = 17 [29.8%]); only 10.6% were the IMPT technique, which spares the esophageal area and heart better with lower toxicity than PSPT. Ho et al. [8] have reported a retrospective analysis of 27 patients with reirradiation of thoracic malignancies using the IMPT technique delivery of a higher dose of radiation (median dose of 66 Gy). Twenty-two patients (81%) were treated for NSCLC. The satisfactory outcomes revealed that patients who received the dose ≥ 66 Gy had increased 1-year freedom rates of local failure (LF) (100% vs 49%; *P* = 0.013), LRF (84% vs 23%; *P* = 0.035), and PFS (76% vs 14%; *P* = 0.050), while no grade ≥ 4 toxicities occurred and only 2 patients (7%) experienced late grade 3 pulmonary toxicity. These studies demonstrate that PBT can provide benefits recurrent NSCLC patients, especially for metastatic lymph nodes in mediastinum, and allow more patients receiving a definitive concurrent chemoradiotherapy.

The feasibility and efficacy of repeated PBT for intrahepatic recurrence or metastasis has been evaluated. Oshiro et al. [9] reported that among the 83 patients with liver cancer who received definitive repeated PBT, the 5-year survival rate of the whole group is nearly 50%, and no patient has radiation-induced liver disease. For reirradiation, it is critical to select the proper patient with the tumor volume and location.

### Pediatric cancers

With more data from children treated with PBT, the proton beam model policy adopted by the American Society of Radiation Oncology in 2017 supports PBT in children with solid neoplasms, and it is now an option for many Children’s Oncology Group (COG) protocols [10]. Many studies have confirmed the feasibility of PBT in pediatric cancer and achieved excellent outcomes compared to photon therapy. The advantage of PBT is recognized for craniospinal irradiation. A phase II clinical study reported the long-term results of PBT in 59 patients (aged 3–21 years) with medulloblastoma [11]. Patients received chemotherapy and had a median craniospinal irradiation dose of 23.4 Gy (RBE) followed by a boost dose of 54 Gy (RBE). The 5-year cumulative incidence of severe hearing loss was 16%. There were no late toxicities of the heart, lungs, and digestive tract side effects, and no second primary tumor occurred, which was significantly better than that of photon therapy; the notable finding was that the intelligence quotient (IQ) of patients using PBT decreased slower than that using photon therapy. The rates of PFS and OS at 5 years were 80% and 83%, respectively. Several studies reported that PBT has been used in the treatment of retinoblastoma, which is a common pediatric intraocular tumor. Mouw et al. [12] reported long-term outcomes for retinoblastoma with PBT. There were no patients died of retinoblastoma or developed metastasis at a median follow-up of 8 years. Eleven of 60 irradiated tumors were enucleated, mainly due to tumor progression. Twelve eyes developed ocular complications requiring intervention, which mainly included cataract, radiation retinopathy, glaucoma, and neovascularization. Various other pediatric cancers including chordoma and chondrosarcoma [13], ependymoma [14], craniopharyngioma [15], low-grade glioma [16], atypical teratoid rhabdoid tumor [17], and Ewing sarcoma [18] were treated with PBT, which is similar in adults, resulting in acceptable toxicities and showing similar survival outcomes to conventional radiotherapy.

With the prolongation of the survival of pediatric cancers, the late response from radiotherapy has received increasing attention. Growing evidence has demonstrated that PBT provide a health outcome benefit in pediatric patients, including radiation-associated late endocrine dysfunction, cognitive ability, and quality of life (QoL). Eaton et al. [19] compared the long-term clinical data in hormone levels after proton and photon irradiation. The results showed that PBT was associated with a reduced risk of hypothyroidism, sex hormone deficiency, and requirement for any endocrine replacement therapy compared to photon therapy, but no significant difference was found in the incidence of growth hormone deficiency, adrenal insufficiency, or precocious puberty. Pulsifer et al. [20] evaluated the cognitive function after PBT in 60 patients with pediatric CNS tumors including medulloblastoma, glioma, craniopharyngioma, ependymoma, and other brain tumors. During the follow-up of 2.5 years, there was a significant decline in the mean processing speed standard score, especially in younger patients (age at baseline < 12 years). The cognitive outcomes compare favorably to published results for patients received photon RT. In a large prospective study, Yock et al. [21] first showed the improved long-term health-related quality of life (HRQoL) outcomes of children with brain tumors treated with PBT compared to photon RT. Leiser et al. [22] reported the QoL were encouraging in children with rhabdomyosarcoma who were treated with pencil-beam scattering (PBS). PBT appears to provide a low risk of second primary tumors, which is a very important problem for pediatric patients treated with RT. Children are in a period of growth and development, with high sensitiveness to radiation, and pediatric patients often have a long-survival time. As mentioned above, in the phase II clinical study [11], patients did not have an occurrence of a second primary tumor during the 7-year follow-up, while a meta-analysis showed the 10-year second tumor and second malignant tumor incidence rates after photon therapy [23] were 6.1% and 3.7%, respectively. Sethi et al. [24] compared the risk of second malignancy in patients with retinoblastoma treated with photon therapy and PBT. At a median follow-up of 13.1 years in the photon therapy group and 6.9 years in the PBT group, the cumulative incidence of second malignancies (radiation-induced or in-field) at 10 years was significantly higher in photon therapy group than that in PBT group (14% vs. 0%; *P* = 0.015). An important challenge in children’s PBT is the anesthesia due to the need of precision therapy. To ensure the precision of repeatability during treatment, most children need anesthesia, which may increase the associated risks.

### Neurological tumor

PBT offers an alternative modality of RT available for neurological tumors in adults, potentially better sparing the surrounding normal brain tissue. Several prospective studies assessed the benefit of PBT in the management of glioma or meningiomas for the patients with low-grade disease, who are usually young with typically long survival with the disease. A proton treatment protocol (NCT01024907) by Maquilan et al. [25] first reported the acute toxicities in patients with low-grade gliomas (LGGs) or meningioma who received 54 Gy. Among the 23 enrolled patients, only 1 patient suffered grade 3 fatigue during the treatment and the follow-up, and only 1 patient had a grade 3 headache at on-treatment visit week 3. There was no observed grade ≥ 3 acute toxicities in a multi-institution prospective study of 58 LGG patients who received PBT with 50.4 Gy to 54 Gy [26]. A study at MGH by Shih et al. [27] showed the findings of 20 LGG patients with the delivered dose of 54 Gy using PBT. The rates of PFS and OS at 5 years were 40% and 84%, respectively. No grade 4 or 5 acute and late side effects occurred. All patients remained stable or slightly improved in neurocognitive status; 6 patients developed hormone deficiency, and there was no significant decrease in quality of life. The side effects of PBT are mild in clinical practice. McDonald et al. [28] reported the results of PBT in patients with World Health Organization (WHO) atypical meningiomas (grade 2). Twenty-two patients received a median dose of 63 Gy (RBE). With the median follow-up of 39 months, the 5-year estimate of LC was 71.1%, and it was 87.5% following a RT dose > 60 Gy (RBE), compared to 50.0% for ≤ 60 Gy (RBE). The data showed that PBT for meningiomas achieved favorable tumor control. For meningiomas that were partially adjacent to vital organs, PBT can be hypofractionated to better control the tumor, which has potential advantages. Vlachogiannis et al. [29] utilized IMPT (4 × 5 Gy or 4 × 6.6 Gy) for treatment of intracranial meningioma (WHO I) in 170 patients, of which 155 were located in the skull base, and reported a 10-year PFS rate of 85%, with 6 patients with pituitary dysfunction, and 5 with signs of radiation necrosis (but only 1 requiring surgery, 5 with visual impairment, and 1 with a tumor cyst). Tumors located in the anterior cranial fossa were significantly increasing the risk of complications.

The preferred treatment of chordoma and chondrosarcoma is surgery. However, chordoma and chondrosarcoma, which originate in the skull base, are difficult to completely resect because the location is close to cranial nerves and blood vessels. To achieve a better local control, the radiation dose should be more than 74 Gy [30]. The treatment efficacy of photon therapy is unsatisfied due to the dose limitation of structures surrounding the tumor, such as the brain stem, temporal lobe and optic nerve, and the radiation dose of the tumor cannot be radical by photon therapy. However, PBT can increase the tumor dose and can better protect normal tissues. PBT has been used for the treatment of radio-resistant chordomas and chondrosarcomas for many decades. The patients with low-grade chondrosarcoma usually have a better long-term survival than those with chordoma in PBT and can even achieve a curable effect. Weber et al. [31] used PBS in 77 patients with skull-base chondrosarcoma. With a median dose of 70 Gy, the actuarial LC and OS rates at 8 years were 89.7% and 93.5%, respectively. Weber et al. have also reported long-term outcomes of skull-base low-grade chondrosarcoma and chordoma patients (*n* = 151) treated with PBS. The rates of 7-year LC were 70.9% and 93.6%, respectively, and the rates of 7-year OS were 72.9% and 94.1%, respectively [32]. The toxicities of PBS for chordoma and chondrosarcoma are mild, which include optic nerve injury, brain necrosis, spinal cord necrosis, and hearing loss. A recent meta-analysis compared the effectiveness of PBT and photon therapy for chordoma [33]. The estimated 10-year OS rates of the PBT group reached 60%, which was significantly higher than that of conventional photon therapy (21%) and SRT (40%). Feuvret et al. [34] reported the results of 159 chondrosarcoma patients treated with either PBT alone or combined with photon therapy. The median dose was 70.2 Gy (RBE) and with a median follow-up of 77 months, the LC and OS rates at 10 years were 93.5% and 87%, respectively. Sixteen patients died, 13 of intercurrent disease and 3 of disease progression. There was no significant correlation between the incidence of toxicity and dose. Spinal cord necrosis is a serious side effect, and a study by Stieb et al. [35] has shown that dose constraints of 64 Gy as a dose to relative volume of 2% (D2%) for the surface spinal cord and 54 Gy for the center spinal cord seemed safe and appropriate for clinical use. Protons have been used in the treatment of functional pituitary adenomas [36], but the data are very limited to date.

### HNC

PBT has been as an option when normal tissue constraints cannot be met by photon-based therapy for tumors of the ethmoid sinus, maxillary sinus, salivary gland, periorbital, nasopharynx, and mucosal melanoma, from the updated 2017 National Comprehensive Cancer Network (NCCN) guidelines. PBT is uniquely suited for HNC with the complex anatomy of tumors and important sensitive OARs, such as brain stem, optic chiasm, and optic nerve. The radiation targets of some HNC, including major salivary gland cancer, skin cancer, early-stage tonsil cancer, and select oral cavity cancer, can be confined to unilateral head and neck, and therefore, lend themselves to the treatment of PSPT, which is better suited to superficial tumors which invade or abut critical structures. Romesser et al. [37] compared the treatment-related toxicities between patients receiving PSPT and IMRT in 41 patients with one side of major salivary gland tumors or cutaneous squamous cell cancers. The results showed that the rates of grade ≥ 2 acute dysgeusia, mucositis, and nausea were significantly lower in PSPT group than those in IMRT group (5.6% vs. 65.2%, 16.7% vs. 52.2%,11.1% vs. 56.5%; *P* < 0.001, < 0.019, = 0.003, respectively). Russo et al. [38] have reported that 54 patients with stage III and IV SCC of the nasal cavity and paranasal sinus received PBT. The median dose was 72.8 Gy (RBE). At 5 years, the PBT yielded good actuarial LC rate of 80%, and the OS rate of 47%. Wound adverse events constituted the most common severe toxicity. Fifteen ≥ grade 3 side effects were observed. No grade 5 toxicity occurred. A meta-analysis study for nasal cavity and paranasal sinus tumors has showed a 5-year locoregional benefit and a slight OS advantage with PBT when compared to IMRT [39]. Decreased acute toxicities such as dysgeusia, mucositis, and nausea occurred in the PSPT group. However, the PSPT group had a higher incidence of grade ≥ 2 dermatitis. Excellent LRC and survival rates were acquired on patients with nasopharyngeal carcinoma (NPC) using PBT. In a phase II trial, Chan et al. [40] assessed the efficacy and side effects of 23 patients with stage III–IVB NPC received concurrent chemo-PBT. With a median follow-up of 28 months, there were no local or regional recurrence occurred, and the 2-year disease-free survival (DFS) and OS were 90% and 100%, respectively. There was no acute or late grade 4 or 5 treatment-related toxicities. A three-dimensional (3D) technique, PSPT with two posterior oblique fields, was used in the study. For treatment of regions in the nasopharynx or oropharynx with the bilateral neck, PSPT seemed to have difficulty achieving high-dose conformality, whereas IMPT has clear dosimetric advantages, providing the ability to cover a large field and deliver the conformity dose to complex head and neck tumors with irregular shapes. Lewis et al. [41] presented the clinical results for 10 patients treated with IMPT. No patients underwent any acute grade ≥ 4 toxicities or any chronic grade ≥ 3 toxicities. With the median follow-up of 24.5 months, 2-year rates of LRC, DMFS, and OS were 100%, 88.9%, and 88.9%, respectively. In a retrospective case-control study [42], IMPT-treated NPC patients (*n* = 10) had significantly lower rates of gastrostomy tube insertion compared to IMRT-treated patients (*n* = 20) (20% vs. 65%, *P* = 0.02). There was no significant difference in chronic grade 3 toxicity, body weight lost, and swallowing dysfunction between type of radiation (*P* = 0.542, 0.333, and 0.175, respectively). No patient developed LF in the IMPT group and 1 did in the IMRT group. One patient in each IMPT and IMRT group developed distant metastatic disease. Additionally, one patient in each group died. A series of studies on patients with (OPC) using IMPT were reported at MD Anderson Cancer Center. Sio et al. [43] retrospectively collected data from a prospective study and discovered that IMPT led to a lower symptom burden during the first 3 months after treatment for OPC patients who treated with IMPT and concurrent chemotherapy. In the same prospective study, Gunn et al. reported the clinical outcome of 50 patients with OPC received IMPT. The encouraging results showed the 2-year OS and PFS of 94.5% and 88.6%, respectively, without grade ≥ 3 acute and late toxicities found [44]. Then, the outcomes of the same cohort from 2011 to 2014 and 100 IMRT OPC patients from 2010 to 2012 were compared [45]. With a median follow-up of 32 months, the significant differences were not found in OS, PFS, acute grade ≥ 3 dermatitis or mucositis between the two groups. The results of the abovementioned comparative studies of IMRT and IMPT in NPC and OPC may be biased due to the case-matched analysis. Additionally, the samples were small in the single-institution case, and the follow-up was relatively short for NPC or OPC patient with favorable OS.

### Eye tumors

Although the incidence of eye tumors is very low, there is a relatively longstanding experience for plenty of patients with eye tumors treated with PBT, yielding excellent survival outcomes with ocular conservation and visual preservation. Lane et al. [46] showed the findings of PBT in 3088 patients with uveal melanoma. With the median follow-up of 12.3 years, the melanoma-related mortality rate was 24.6% in 15 years after treatment and 26.4% in 25 years. The highest annual rates of death from melanoma were reported 3 to 6 years after PBT, with the death rates of approximately 3–4%. A study of 982 patients with uveal melanoma treated with PBT showed that the 10-year LC and overall eye retention rates were 96.4% and 95%, respectively, with a median follow-up of 60.7 months [47]. The toxicities were acceptable, where 115 (12.1%) patients developed glaucoma and 30 patients had to be enucleated. In a retrospective study that enrolled 336 patients with large choroidal melanomas, the rates of visual acuity retention at 10 years were 8.7% for ≥ 20/200 and 22.4% for at least counting fingers; neovascular glaucoma was found in 25.3% patients. The rates of eye retained and tumor controlled were 70.4% and 87.5%, respectively, at 10 years post-PBI therapy. The 10-year rates of all-cause mortality and dying of metastatic uveal melanoma were 60.7% and 48.5%, respectively [48]. Verma et al. [49] reviewed the results of 14 studies of PBT for uveal melanoma, which was consistent with prior studies. In a retrospective study with 492 choroidal melanomas patients receiving PBT [50], the 5-year LC was high at 94%, and the survival was not deteriorative. The mean baseline visual acuity, visual acuity ≥ 20/200, neovascular glaucoma, and enucleation were in 31.7% (20/63), 20%, 27%, and 19.5%, respectively. The study indicated that PBT was a safe strategy for large choroidal melanomas. Similarly, in order to achieve good vision function and cosmesis, PBT is an attractive RT mode for patients with periorbital tumors. At MD Anderson Cancer Center, 20 patients with lacrimal gland (*n* = 7), lacrimal sac/nasolacrimal duct (*n* = 10), and eyelid (*n* = 3) underwent orbit-sparing surgery followed by PBT [51]. With a median follow-up of 27.1 months, no patient had local recurrence, only 1 suffered regional recurrence and another 1 distant metastasis. There were no patients who experienced acute grade 3 ocular disorders, acute and chronic grade ≥ 4 toxicity. Meanwhile, the good local control has been obtained [52]. Among 11 patients who experienced orbit-sparing surgical resection followed by PBT and/or chemotherapy, 10 patients had post-treatment visual acuities better than 20/40 and were also satisfied with their cosmesis after eye-sparing surgery. PBT achieved good LC and was well tolerated with a good vision function and cosmesis.

The eye toxicities were acceptable for patients treated with PBT. Thariat et al. [53] showed the 5-year incidence of dry-eye syndrome and severe (grade 2–3) dry-eye syndrome was 23.0% and 10.9%, respectively. Patients whose tumors located on the superotemporal or temporal lobe had a higher risk for severe dry-eye syndrome.

The lens is one of the most radiosensitive organs and can cause cataracts when exposed. PBT can better spare all or part of the lens than other forms of RT. The 5-year incidence of cataract was 18.7%, and the corresponding vision-impairing cataract rate was 12.8% of 1696 ocular melanomas by PBT [54]. For tumors which are located on the upper side of the choroid plexus, if the upper eyelid margin is not retracted out of the radiation field, patients abrade the cornea every time they are blinking. This may cause keratopathy, and it can become so severe as to cause corneal enucleation. However, transpalpebral (i.e., through closed eyelids) PBT of choroidal melanoma can spare the eyelid and avoid ocular surface complications without increasing failure of local control [55].

### NSCLC

The toxicity of cardiopulmonary, lung, and spinal cord restricts the ascent of dose for patients with NSCLC by RT with or without chemotherapy. PBT’s early use in NSCLC was confined to small (stage I) tumors with conventional fraction, producing a high rate of LC. For stage I NSCLC, it is interesting in stereotactic body proton radiotherapy (SBPT). Loma Linda University reported clinical experiences in the early-stage NSCLC (*n* = 111) with SBPT [56]. With the dose escalated from 51 Gy to 70 Gy in 10 fractions, the OS was improved, with a 4-year OS rate of 18% up to 51% (*P* = 0.006). Chang et al. [57] have reported a modified less hypofractionated regimen of PBT with a total dose of 87.5 Gy and 2.5 Gy per fraction in 35 early-stage NSCLC patients. 5-year rates of local recurrence-free, regional recurrence-free, and DMFS were 85.0%, 89.2%, and 54.4%, respectively. On the basis of the encouraging results, MD Anderson Cancer initiated a phase II randomized trial of SBRT (*n* = 9) vs. SBPT (*n* = 10) in stage I–II or recurrent NSCLC [58]. Unfortunately, similar 3-year LC rates were reported, at 87.5% and 90% in these two groups, respectively. Larger cohort studies are needed regarding the safety and efficacy of SBPT in comparison to SBRT. Based on the dosimetric advantage, PBT has the potential to escalate the higher dose within target.

For patients with locally advanced NSCLC who received a high proton dose with or without chemotherapy have been reported. A retrospective study reported 35 patients with stage II–III NSCLC receiving PSPT [59]. With a mean dose of 78.3 Gy (RBE), 2-year local PFS was 65.9% and OS rate was 58.9%. Severe toxicity was not observed. In a non-randomized prospective study [60], 134 NSCLC patients with stage II (*n* = 21) and stage III (*n* = 113) underwent PSPT concurrent with weekly chemotherapy. The rates of grade 3 and grade 4 toxicities were 12% and 0.7%, respectively. This study demonstrated that a high proton dose of 60–74.1 Gy (RBE) was safe and tolerable with low toxicity. The median OS were 40.4 and 30.4 months for patients with stage II and stage III, respectively, and the promising 5-year OS rates was 25.3% for stage IIIA and 31.8% for stage IIIB. The results suggested that patients with larger tumors and centrally located lesions or those near the brachial plexus may be of benefit more with the use of PBS. Recently, Chang et al. [61] provided a phase II study which described the final outcome of concurrent chemotherapy and PSPT with 74 Gy for unresectable stage III NSCLC (*n* = 64). With a median follow-up of 27.3 months, the results showed favorable outcomes OS of 29%, and PFS of 22% at 5 years. There was no acute or late grade 5 toxicity. The rate of three acute esophagitis was 5 (8%). Late toxicities were not common: 1 patient experienced grade 3, 1 grade 4 esophagitis, 8 grade 3 pneumonitis, 1 grade 4 bronchial fistula, and 2 grade 3 pericardial effusions. This is consistent with prior phase II studies, which indicated that concurrent a high proton dose and chemotherapy was well tolerated and effective for stage III NSCLC [62, 63]. Patients with locally advanced NSCLC received a high proton RT dose had excellent outcomes with tolerable toxicity. To confirm whether PBT could benefit local disease control and survival, Liao et al. conducted the first one randomized trial comparing PSPT (*n* = 57) with IMPT (*n* = 92) for patients with locally advanced NSCLC received concurrent chemotherapy [64]. Unfortunately, the significant difference was not observed in the grade ≥ 3 radiation pneumonitis (IMRT vs. PSPT: 6.5% vs. 10.5%; *P* = 0.537) or local failure (IMRT vs. PSPT: 10.9% vs. 10.5%; *P* = 1.0) after IMRT or PSPT. It should be noted that these above studies used PSPT, which may restrict the advantage of protons. Phase III trials (RTOG 1308) using IMPT with 70 Gy (RBE) vs. IMRT are ongoing [65]. The results may reveal whether PBT benefit the patients with advanced NSCLC or not.

### Breast cancer

The clinical experiences with PBT for patients with breast cancer are limited, and fewer studies have centered on accessing the clinical outcomes of long-term follow-up. At first, studies using PBT for breast cancer focused on accelerated partial breast irradiation (APBI), where recurrence risk was low and treatment-related toxicity was less tolerable. One of the largest APBI study by Bush et al. [66] was reported with 40 Gy (RBE) in 10 daily fractions in 100 patients. With a median follow-up of 60 months, cosmesis was good to excellent in 90% patients, grade ≥ 3 acute skin reaction was not occurred, yielding DFS and OS of 94% and 95%, respectively. PBT is also a promising mode for adjuvant radiotherapy in breast cancer with nodal areas. Verma et al. [67] reported acute toxicities in 91 patients who had adjuvant breast/chest wall and regional nodal radiotherapy using PBS or PSPT with a median dose of 50.4 Gy (RBE). The median follow-up was 15.5 months. Grades 1, 2, and 3 dermatitis occurred in 23%, 72%, and 5% of patients, respectively, and grades 1, 2, and 3 esophagitis arose in 31%, 33%, and 0%, respectively. There are some studies that have reported the acute toxicities of PBT for patients treated with postoperative RT [68, 69]. Although the potential for PBT to prevent cardiac deaths is dosimetrically apparent [70], it needed to further evaluate whether PBT could actually reduce late cardiac toxicity due to the short of long-term follow-up data.

### Esophageal cancer (EC)

Currently, IMRT is the most common radiation technique in treating EC. To date, the clinical experience of PBT for patients with EC has lack of institutional studies. Ishikawa et al. [71] performed definitive PBT and concurrent chemotherapy in 40 patients with esophageal squamous cell carcinoma. Patients received a total dose of 60 Gy (RBE), and an additional boost of 4–10 Gy (RBE) was given when residual tumors were suspected. There was no grade ≥ 3 cardiopulmonary toxicities. The 3-year rate of OS was 70%, and 2-year rates of DFS and LRC were 77% and 66%, respectively. Compared with squamous cell carcinoma, patients of adenocarcinoma had inferior outcomes; the 3-year rates of OS, relapse-free survival (RFS), DMFS, and LRF survival were 51.7%, 40.5%, 66.7%, and 56.5%, respectively. Recently, Prayongrat et al. [72] have reported excellent clinical outcomes of 19 patients with EC treated with concurrent chemo-radiotherapy using PBS. The median doses were 50.4 Gy (RBE) in 28 fractions. With a median follow-up time of 17 months, the OS was 39.2 months. The 1-year rates of OS, locoregional RFS, and DMFS were 100%, 88.8%, and 72.9%, respectively. Treatment was well tolerated with limited grade 3 toxicities. Clinically complete response was achieved in 84% of patients. Grade 3 esophagitis and fatigue occurred in three patients, and grade 3 esophageal strictures occurred in only 1 patient. The clinical outcomes of PBT combined with chemotherapy for EC were encouraging in the above studies. The comparison of clinical outcomes between proton and photon RT has only been reported in one retrospective study [73]. From 2007 to 2014, 343 EC patients treated with definitive chemo-radiotherapy were enrolled. Compared with IMRT (*n* = 211), PBT (*n* = 132) had significantly better OS, PFS, and DMFS (*P* = 0.011, 0.001, 0.031, respectively), as well as marginally better LRFFS (*P* = 0.075). However, there was no significant difference in treatment-related toxicities rates between two groups. In the PBT group, most patients (94.7%) received PSPT, and only 5.3% patients (7) were treated with IMPT. Subgroup analysis by clinical stage found significantly higher rates of OS (34.6% vs 25.0%, *P* = 0.038) and PFS (33.5% vs 13.2%, *P* = 0.005) at 5 years in the PBT group for stage III patients, but no significant differences in intergroup survival were observed for patients with stage I/II. The findings suggested that the theoretical advantage of PBT over photon therapy might turn into a survival benefit, especially in locally advanced disease.

Recently, one notable study at MD Anderson was reported that grade 4 lymphopenia during chemo-radiotherapy for EC was associated with poor overall and disease-specific survival outcomes, and OS in this group was significantly worse than the grade 0–2 group, with a median OS 2.8 vs. 5.0 years (*P* = 0.027) [74]. The radiation type (photon-based VS. proton-based) significantly influenced the mean body dose exposure, which was a strong predictor for G4 nadir (*P* < 0.01). The important finding in the study was that PBT could reduce the low dose area, and then resulted in less lymphopenia risk. The study revealed that PBT could help to improve immune surveillance, and better tumor control may finally be a benefit from it. The critical role of protons for immune surveillance requires confirmation in further research.

### Liver cancer

The tolerated dose of normal liver is relatively low, and 80% of patients with liver cancer have chronic liver disease, which further reduces the tolerated dose of normal liver. Although liver cancer cells are highly sensitive to radiation, the usage of photon RT is limited for liver cancer. However, PBT can significantly decrease the normal liver dose, and most of the normal liver can be completely unirradiated, which makes it possible to use dose escalation. A phase I study suggested that 72 GyE in 24 fractions using PBT for patients with inoperable hepatocellular carcinoma (HCC) was safe and effective with a complete response (CR) rate of 100%, 3-year local PFS rate of 83.3%, DFS rate of 20.8%, and OS rate of 73.3% [75]. Hong et al. [76] showed a multi-center phase II clinical study of high-dose, hypofractionated PBT for localized inoperable liver cancer. There were 83 patients enrolled. With a medium dose of 58 Gy/15F, the median diameters of HCC and intrahepatic cholangio carcinoma were 5.0 cm and 6.0 cm, respectively, of which 27.3% and 12.8% were multi-centric, and 29.5% and 28.2% had tumor vascular thrombosis. The rates of LC at 2 years were 94.8% and 94.1%, and the rates of OS at 2 years were 63.2% and 46.5%. The most common toxicities were fatigue, rash, nausea, or anorexia. Four patients had grade ≥ 3 side effects: liver failure and ascites, thrombocytopenia, gastric ulcer, and elevated bilirubin. Recently, similar LC and OS of HCC over 5 cm after PBT (median dose of 72.6 Gy in 22 fractions) in 24 patients were reported by offering an effective and safe RT that yielded a 2-year LC and OS rate of 87% and 52.4% for 24 patients with HCC over 5 cm [77]. Bush et al. [78] compared the effects of PBT and transcatheter arterial chemoembolization for liver cancer. There was a trend toward improved 2-year LC (88% vs. 45%, *P* = 0.06) and PFS (48% vs. 31%, *P* = 0.06) favoring the PBT group and significantly fewer hospitalization days were found in the PBT group. The data of long-term efficacy of PBT for patients with untreated HCC is limited. Fukuda et al. [79] reported the 5-year outcomes for 129 patients. Total PBT dose was 66.0~77.0 GyE in 10~35 fractions, the rates of LC, PFS, and OS at 5 years were 94%, 28%, and 69% for 0/A stage patients (*n =* 9/21), 87%, 23%, and 66% for patients with B stage (*n =* 34), and 75%, 9%, and 25% for those with C stage (*n =* 65), respectively. For 15 patients with tumor thrombi in major vessels, the rates of LC and OS at 5 years were 90% and 34%, respectively. There was no grade ≥ 3 toxicity. PBT offered an effective and safe therapy for HCC patients with portal vein tumor thrombosis, which has limited treatment options. With a median dose of 55 Gy PBT at 20~22 fractions, a promising result was median OS of 13.2 months, the partial response of 55.6% (15/27), stable disease of 37% (10/27), and progressive disease of 7.4% (2/27). There was no toxicity of grade ≥ 3. PBT is a promising RT modality to treat cancer thrombosis, which is the common complication for liver cancer with poor prognosis. With the high-dose PBT, more than 50% of tumor thrombosis can be alleviated and then significantly prolong the survival time of patients [80]. With the development of technology, the application of IMPT may further reduce the dose of normal liver, especially when the tumor is larger and deeper. However, when the tumor is close to the chest wall, the chest wall toxicity risk cannot be avoided without sacrificing the tumor coverage, and it may be reduced with continuously IMPT optimization [81].

### Prostate cancer

PBT is the most widely used in the treatment of prostate cancer. Takagi et al. [82] reported the clinical outcomes in patients with limited stage prostate cancer received PSPT, which had the largest cohort of patients (*n* = 1375) and the longest follow-up period to date. The conventional fractionation was used, and 99% of patients treated with 74 Gy (RBE). With a median follow-up of 70 months, for the low-, intermediate-, high-, and very high-risk groups, 8-year freedom from biochemical relapses were 95%, 87%, 71%, and 55%, respectively, and 8-year cancer-specific survival rates were 100%, 99%, 98%, and 92%, respectively. The findings revealed that the incidence of late genitourinary toxicity continued to increase beyond 5 years, whereas the incidence of late gastrointestinal toxicity had plateaued by 5 years. Similar results were reviewed in 1327 patients by Bryant et al. [83]. Ho et al. [84] evaluated long-term outcomes with a focus on sexual health for young patients treated with PSPT in a dose of 76–82 Gy (2 Gy/F) or 70–72.5 Gy (2.5 Gy/F). The results were shown that erections firm enough for sexual intercourse decreased from 90% (baseline) to 72% (1 year follow-up). Only 2% of patients underwent urinary incontinence with pads. The bowel habits mean score decreased from 96 at the baseline level to 88 at 1-year follow-up, but it increased to 93 at 5-year follow-up. The clinical outcomes of patients treated with PBT are superior to those treated with three-dimensional conformal radiation therapy photon, which were in other studies. To date, there are no prospective trials comparing the effectiveness and toxicities between proton and photon RT for patients with prostate cancer.

Hypofractionated PBT has been studied in prostate cancer and is expected to become an effective treatment approach. Henderson et al. [85] showed the results that the accelerated hypofractionated regimen for low-risk and intermediate-risk prostate cancer with 2.5 Gy per fraction; the 5-year OS rates were 96% and 96.4%, respectively, while the 5-year freedom from biochemical relapses were 98.3% and 92.7%, respectively. The actuarial 5-year rate of late radiation-related ≥ grade 3 gastrointestinal side effect was 0.5%, and urologic toxicity was 1.7%, which showed the hypofractionated regimen had high efficacy and was well-tolerated. Nakajima et al. [86] compared the differences in acute toxicity among patients with intermediate- and high-risk prostate cancer received conventional fractionated PBT (2Gy/F) and the hypofractionated regimen (3 Gy/F). No severe acute side effect occurred in either group. Grade 2 acute genitourinary toxicities rates were 15% (*n* = 38) in the conventional fractionated group and 5.9% (*n* = 16) in the hypofractionated group (*P* ≤ 0.001), but no significant differences in acute gastrointestinal toxicity were found between both groups. The interim results of the PCG GU 002 trial showed that the hypofractionated regimen of 38 Gy RBE (7.5 Gy RBE/fraction) for low-risk prostate cancer patients was tolerated well, with no grade ≥ 3 acute toxicity, and it revealed no apparent clinical difference in outcomes compared with conventional fractionation [87]. To reduce the rectal dose and toxicity, Chung et al. [88] inserted a spacer in the prerectal space and the thickness of the spacer was no less than 9 mm to yield the largest benefit. For prostate cancer treated with PBT, it is important to emphasize that patients with hip or femoral head replacement were not suitable for using two horizontal beams through the opposing right and left lateral femoral head, which is usually designed in IMPT planning. An alternative dose delivery technique is with two anterior-oblique beams, whereas it could increase the dose exposure to the rectum [89].

## The current challenges of proton therapy and its development in the future

Growing application of PBT to treat patients with malignancy has been confirmed to be safe, precise, and efficient with a tolerant toxicity, resulting in expanding the clinical applications in spite of that the vast costs and building sites are required to install and maintain the PBT treatment machine. During the last decade, the proton facilities are most widely distributed worldwide. As of August 2018, there were approximately 70 proton centers in operation in the world, and 45 were under construction; more than 140,000 patients have been treated by PBT [90, 91]. The statistics of proton centers and patient treated by PBT are shown in Table 1. As of November 13, 2017, there were approximately 300 clinical trials with PBT that are ongoing, and the detail is shown in Table 2 [92]. However, there are at least three limitations of published studies that evaluate the value of PBT. First, most studies were retrospective analyses. Second, the prospective studies had small samples. Last, the data for comparisons between PBT and conventional RT were limited. Further prospective trials with modern techniques should be more valuable to confirm whether the advantage of protons can be transferred into a benefit for clinical outcome and late effects in HNC.

**Table 1:** Facilities in operation patient statistics (last update August 2018) and facilities under construction (update July 2018)

Status	Area	Country/region	Numbers of proton centers	Total patients treated
Operation	Asia	China	2	1729
		Japan	13	23,035
		South Korea	2	2056
		Taiwan, China	1	1010
	Europe	Czech Republic	1	2428
		England	2	3224
		France	3	14,881
		Germany	6	9752
		Italy	3	1302
		Poland	1	267
		Russia	3	5552
		Sweden	1	407
		Switzerland	1	8448
		The Netherlands	1	1
	North America	USA	27	72,009
		Canada	1	204
	Oceania	Australia	1	79
	Africa	South Africa	1	524
		Total	70	149,086
Under construction	Asia	China	7	
		Japan	5	
		Thailand	1	
		South Korea	1	
		India	2	
		Emirate of Abu Dhabi	1	
		Singapore	1	
		Taiwan China	2	
		Saudi Arabia	1	
	Europe	France	1	
		The Netherlands	2	
		Russia	2	
		UK	6	
		Denmark	1	
		Belgium	1	
		Slovak Republic	1	
	North America	USA	10	
		Total	45

**Table 2:** Clinical trials for proton beam therapy (update November 13, 2017)

Indication:	Loc:	Links to protocols (clinicaltrials.gov and UMIN-CTR):
Pediatrics	Craniopharyngioma	NCT01419067; NCT02792582
	Central nervous system tumors	NCT02559752; NCT01180881; NCT02112617
	Brain tumors	NCT00602667; NCT01288235; NCT01115777; NCT00105560; NCT03267836; NCT00238264; NCT03281889
	Head/neck	NCT02608762
	Bone	NCT00592293
	Rhabdomyosarcoma	NCT00592592
	Lymphoma involving mediastinum	NCT01751412
	Unclassified	NCT01502150; NCT02644993; NCT03223766; NCT01696721; UMIN000023170
Head and neck	Nasopharynx	NCT00592501; NCT01586767; NCT03274414
	Oropharynx	NCT01893307; NCT02663583; NCT02736786
	Esophageal	NCT01512589
	Unclassified	NCT01228448; NCT01627093; NCT01973179; NCT02838602; NCT02923570; NCT03183271
Lung	Non-small cell lung cancer	NCT00614484; NCT01511081; NCT00495040; NCT01512589; NCT01165658; NCT00915005; CT01808677; NCT00875901; NCT00881712; NCT01770418; NCT02029222; NCT02038413; NCT02844140; NCT01629498; NCT01993810; NCT01076231; NCT01108666; NCT01126476; NCT02130427; NCT03087760; NCT01525446; NCT01565772; NCT02314364; NCT02204761; NCT02172846; NCT02172846; NCT02073968; NCT01859650; NCT02731001; UMIN000005585; NCT03132532; NCT03226925
CNS	Brain tumors	NCT01854554; NCT01730950; NCT02179086; NCT01024907; NCT01180881; NCT0135805; NCT01228448; NCT0328633; NCT02693990; NCT03286335; NCT01165671; NCT02607397; NCT01730950; NCT02824731; NCT02824731; NCT03180502; NCT03281889; NCT01117844; NCT01180881; NCT00798057
	Skull base	NCT01795300; NCT01182753; NCT01182779
	Chondrosarcoma	NCT00496522
	Central nervous system	NCT01049230; NCT02559752; NCT02797366; NCT03055364
Breast	Partial breast	NCT01839838; NCT01386697; NCT00599989; NCT02603341; NCT02199366; NCT02725840; NCT01340495; NCT03270072; NCT03340402; NCT00614172; NCT01310530; NCT01766297; NCT01758445; NCT01245712; NCT02453737; NCT03339934; UMIN000017579; UMIN000016206
	Lymph nodes	NCT02783690; NCT01365845
GI	Liver	NCT00614913; NCT01141478; NCT00857805; NCT01697371; NCT00976898; NCT00465023; NCT01239381; NCT00662246; NCT01963429; NCT01643824; NCT02395523; NCT00426829; NCT01668134; NCT02632864; NCT02571946; NCT02640924; UMIN000020596; NCT02802124; UMIN000020862; UMIN000002863; UMIN000025342; UMIN000020596; UMIN000016574; NCT03186898
	Pancreas	NCT01821729; NCT01591733; NCT00438256; NCT01494155; NCT00658801; NCT00658840; NCT00685763; NCT00763516; NCT01553019; NCT02598349; NCT01683422; UMIN000020862; UMIN000008785; UMIN000012201
	Upper GI	NCT01449864
	Rectum	NCT00503932; NCT03018418; NCT03098108
	Esophageal	NCT01512589; NCT01684904; NCT02023541; UMIN000015550; NCT03234842
GU	Prostate	NCT02110849; NCT01709253; NCT03285815; NCT01811810; NCT01352429; NCT01045226; NCT01617161; NCT02040610; NCT00969111; NCT00693238; NCT01368055; NCT01072513); NCT01040624; NCT01987193; NCT02598349; NCT00489814; NCT01950351; NCT00388804; NCT01492972; NCT01603420; NCT01230866; UMIN000020199; UMIN000010510; UMIN000017679; UMIN000017679; UMIN000020596; UMIN000003937; NCT02766686; NCT02874014
	Bladder	NCT01520038
Lymphoma	Hodgkin lymphoma	NCT02070393; NCT00850200; NCT02404818; NCT01751412
Sarcoma	Chordoma, chondrosarcoma	NCT00797602; NCT00881595; NCT00901836; NCT0049652; NCT00496119; NCT01449149; NCT01561495; NCT01182753; NCT01904565; NCT01819831
	Spine	NCT01346124; NCT00592345
	Retroperitoneal	NCT01659203; NCT01034566
	Sacrococcygeal	NCT01811394; NCT02986516
Female reproductive system	Rhabdomyosarcoma Cervical and endometrial	NCT01871766 NCT03184350

*Abbreviations*: *CNS* central nervous system, *GI* gastrointestinal, *GU* genitourinary

Besides, there are currently still some great challenges in the precision PBT. In addition, in the future, there will be more advances in precision proton radiotherapy to benefit more patients.

### Technical developments in precision proton radiotherapy

The proton planning system and facility are advanced, which makes PBT increasingly precise over time. The target volume is usually larger than the high-dose covered by the Bragg peak. Spread-out Bragg peaks (SOBP) are needed to make sure every tissue element in the target receives the same amount of dose. In the early days, the dose mainly delivered by PSPT used the beam double scattering and range modulation techniques. To spare the normal tissues in the lateral and distal tumor, the aperture and range compensator are usually needed. The drawbacks of scattering technique include broadened lateral penumbra, secondary particles, e.g., neutrons, from the scatters, and need for the numerous pieces of hardware for every treatment field. With the advanced development of computers and technology, the active scanning technique, named IMPT, including intensity-modulated scanning, PBS, and spot scanning, can overcome the drawbacks of the scattering system, obtain better dose conformity, and reduce the integral non-target dose. However, the active scanning technology is very sensitive to organ motion and change, because it delivers the dose to different parts of the target sequentially. Therefore, it is required that the boundary, motion, and changes of GTV and OARs are accurately determined. Meanwhile, the equipment with protons is more advanced with time, which is also very important for precision PBT. In the earliest proton facilities, the beam was fixed in 1 to 2 directions was fixed. To some extent, the restrictions of fixed beam, beam energy, and field size in turn limit the advantage of protons. Currently, most newly constructed facilities have 360° rotational gantries that allow treatment of tumors at any anatomic site, and the therapy system has the IMPT planning capabilities.

To fully take advantage of the depth-dose benefit, it is more important to define the range of the proton beam as accurately as possible. The range uncertainty in patients mainly arises from CT imaging and calibration, CT resolution, and CT Hounsfield units (HU) to relative stopping power (RSP) conversion [93]. To improve the accuracy of the proton beam range, more advanced devices including simulation MRI, dual-energy CT, and proton CT can be used. The current single-energy CT leads to related uncertainties in the proton range of approximately 3%. To ensure the target received the prescription dose, the range uncertainty should be included, which will lead to the normal tissues around target receiving much more radiation dose. Recently, studies have focused on reducing the range uncertainty and improving its accuracy, and the dual-energy CT was suggested to be used in the proton therapy. Previous studies have reported that dual-energy CT potentially improved the conversion from CT HU to RSP, which could reduce the proton beam range uncertainties by 0.4% in soft tissues, and reduce the RSP uncertainty from 1.59% to 0.61% for homogeneous tissue-equivalent [94, 95]. However, the dual-energy CT only reduces uncertainty arising from the conversion of CT HU to RSP but cannot eliminate it. Several studies have demonstrated that the proton CT, whose image-formation characteristics are based on the linear stopping power of protons, avoids the uncertainties of mapping x-CT HU values to RSP [96]. Arbor et al. [97] has validated the proton CT benefit based on a Monte Carlo comparison. Studies have demonstrated that the proton CT has the potential to outperform the accuracy achievable with dual energy CT [98, 99]. Another potential advantage of the proton CT is that it needs fewer doses to achieve the same quality image [100]. This kind of proton CT device is still currently in development and has not been used in clinical settings.

It is a great challenge to precisely calculate the dose in a treatment planning system. There are mainly two methods to calculate the dose: analytical algorithms and the Monte Carlo method. The accurate calculation dose of the Monte Carlo method is much higher than the former, which is a common method to use at present. Previous studies have analyzed the differences between analytical algorithms and Monte Carlo dose computations in proton therapy [101]. Urie et al. [102] has investigated that the analytical algorithms could not able to precisely predict the effect of range degradation, due to the fact that it is less sensitive to complex geometries and density variations. The study has compared Monte Carlo dose with analytical dose computations based on 525 patients, and found that the analytical method overestimates the dose in the tumor target by nearly 10%; however, the dose in some OAR could be underestimated about 10 Gy [103]. It has the potential to increase some toxicities. Monte Carlo algorithms should be applied to accurately calculate the dose to improve target coverage and spare the OAR in PBT. Currently, only a few proton centers use the Monte Carlo algorithms. It needs more time to compute the dose, which limits the application in clinical settings. However, with the development of computers, it would take much less time for Monte Carlo computation.

### The effect of anatomical changes in precision proton radiotherapy

The effect of dose distribution caused by anatomical changes in proton therapy is more sensitive than photon therapy. Therefore, it is very critical to delineate accurately the GTV and monitor motion and changes of GTV and OARs. Apart from training physicians for GTV and OARs delineation with precision, there are several techniques to reduce the effect of dose distribution by anatomical changes. First, MRI can provide more detailed anatomical boundaries for GTV compared with CT images, including NPC, liver cancer, and colorectal cancer. Schmidt et al. [104] reviewed that MRI could apply to wide range of image contrast mechanisms and use to RT treatment planning. In addition, a number of challenges are reviewed: the effects of patient motion during the long-time scan, an estimate of electron density for tissues, MRI is acquired in the radiotherapy treatment position, and the geometrical accuracy. Second, for patients with lung cancer or liver cancer, the tumor movement during treatment with the breath is more significant. To keep the tumor receiving the prescribed dose, anatomic motion management strategies are currently used in proton therapy including respiration gating [105], real-time tumor tracking [106], and breathe and hold techniques [107]. Breathe hold techniques provide a relatively stable breath in phase of radiation therapy, which minimize the breath motion effect. However, patients need to have a better pulmonary function for the technique. Third, periodic imaging in the course of treatment is used to monitor and assess the changes in patient anatomy generated by tissue deformation, tumor shrinkage, weight loss, and so on. Kraanet al. [108] concluded that bulky radiosensitive human papillomavirus-positive tumors and cervical lymph nodes can respond early in the therapy course causing considerable anatomical changes, which might contribute to a less predictable proton dose distribution. It is not clear whether the treatment plan needs to be reformulated. Image-guided radiation therapy (IGRT) [109], cone beam CT (CBCT) or orbital CT (CT-on rail) is usually used to conduct an image scan before each irradiation for photon therapy. However, it has not widely been used in proton centers. Regular CT scanning is used in some studies. However, the optimum internal time of repeated CT scanning has not been defined, and the tracking technique or repeated CT scan causes the patient’s exposure to ionizing radiation. Last, adaptive radiotherapy is a promising way to adjust the radiation dose distribution according to the changes of tumors and OARs [110].

### Biological effectiveness in precision proton radiotherapy

The RT treatment planning is made on the basis of the prescription doses to the target and constraints for normal tissues. Proton treatment planning is currently planned and delivered assuming a proton relative biological effectiveness (RBE) relative to photons of 1.1 [111], which has usually been used. To date, there is very different comprehension of the 1.1 of RBE. Some studies considered that 1.1 of RBE were acceptable in clinical settings, which was an averaged value of measured RBE, neglecting any dependency of RBE on dose, endpoint or proton beam properties. Others disagree that 1.1 of RBE is an invariable value. In particular, the distal edge of the proton SOBP should be given much attention. The RBE quickly increases as the sharply increasing LET, which will underestimate the effectiveness in the surrounding tissue, causing more unexpected toxicity or complication. In a retrospective subset analysis, patients with oligodendroglioma treated with proton RT developed pseudoprogression earlier compared to photon therapy (48 days versus 131 days). However, there was no difference in those with astrocytoma. The finding suggests the biological effect of proton radiation is different between oligodendroglioma and astrocytoma [112]. Moreover, it is a great challenge to precisely measure the RBE value for the desired position due to the sharp distal fall-off of SOBP. Wouters et al. [113] has investigated the depth and dose dependence of RBE. In addition, the averaged RBE value for entrance, proximal half, distal half, and distal edge was 1.07, 1.1, 1.17, and 1.21, respectively, and the RBE was determined to have dose dependence. Maeda et al. [114] have evaluated the RBE of the spot-scanning beam in different depth of SOBP and found that the distal region showed higher RBE values; these results are in line with those previous studies conducted using PSPT. A study by Jones et al. [115] has demonstrated that the widest RBE ranges existed in low α/β value biosystems because of dose per fraction varies and improving linear energy transfer (LET), usually exceed 1.1 even within the SOBP LET range, with lower RBE values at higher dose per fraction. For tumors with greatly radiosensitive, the RBE values are usually less than 1.1 and insensitivity to per fraction. Therefore, it is important to reduce the LET in normal tissue due to the fact that RBEs increase with LET. However, all the results were based on the in vitro and animal systems [116]. There are limited published clinical data that would investigate the effectiveness for certain tumors or OARs. To the best of our knowledge, there is only one study by Zhang et al., only in a meeting abstract [117]. It attempted to find the end-of-range RBE in the temporal lobe based on long-term follow-up data from patients with NPC. The findings showed that the brain-specific end-of-range RBE could be ≥ 1.8, 7.3% higher than what is currently used in clinical settings. The optimal RBE has not been defined. RBE may be different in different biological diseases. The RBE varying with LET, physiological and biological factors, and clinical endpoints still requires further research.

## Conclusions

The dosimetric advantage of protons results in a finite range with little or no exit dose and a smaller volume of normal tissue to be irradiated. It is worth noting that the precision is becoming increasingly more important to take advantage of PBT for patients. The technical advances allow that the precision PBT will become widely available, and it may be the lead application in the treatment of cancer in the future. Optimization of the PBT, appropriate integration of the proton beam with chemotherapy, target therapy, biological therapy, or immunotherapy, would further benefit patients with aggressive tumors, providing excellent survival and less toxicity.

## Abbreviations

APBI: Accelerated partial breast irradiation
CBCT: Cone beam CT
COG: Children’s Oncology Group
CR: Complete response
DFS: Disease-free survival
DMFS: Distant metastasis-free survival
EC: Esophageal cancer
FDM: Freedom of distant metastasis
HCC: Hepatocellular carcinoma
HNC: Head and neck cancer
HRQoL: Health-related quality of life
HU: Hounsfield units
IGRT: Image-guided radiation therapy
IMPT: Intensity-modulated proton therapy
IMRT: Intensity-modulated radiotherapy
IQ: Intelligence quotient
LET: Linear energy transfer
LGG: Low-grade glioma
LRC: Locoregional control
LRF: Locoregional failure
LRFFS: Locoregional failure-free survival
MGH: Massachusetts General Hospital
NCCN: Comprehensive Cancer Network
NPC: Nasopharyngeal carcinoma
NSCLC: Non-small cell lung cancer
OARs: Organs at risk
OS: Overall survival
PBS: Pencil-beam scattering
PBT: Proton beam therapy
PFS: Progression-free survival
PSPT: Passive scatter proton therapy
QoL: Quality of life
RFS: Relapse-free survival
RSP: Relative stopping power
RT: Radiotherapy
SBPT: Stereotactic body proton radiotherapy
SOBP: Spread-out Bragg peaks
WHO: World Health Organization
